# Cross-sectional associations between neighborhood characteristics, cognition and dementia risk factor burden in middle-aged and older Australians

**DOI:** 10.1016/j.pmedr.2024.102696

**Published:** 2024-03-19

**Authors:** Marina G. Cavuoto, Liam Davies, Ella Rowsthorn, Lachlan G. Cribb, Stephanie R. Yiallourou, Nawaf Yassi, Paul Maruff, Yen Ying Lim, Matthew P. Pase

**Affiliations:** a Turner Institute for Brain and Mental Health, School of Psychological Sciences, Monash University, Melbourne, Australia; b National Ageing Research Institute, Royal Melbourne Hospital, VIC, Australia; c Centre for Urban Research, School of Global, Urban and Social Studies, RMIT University, City Campus, Melbourne, Victoria, Australia; d Department of Medicine and Neurology, Melbourne Brain Centre at The Royal Melbourne Hospital, University of Melbourne, Parkville, VIC, Australia; e Population Health and Immunity Division, The Walter and Eliza Hall Institute of Medical Research, Parkville, VIC, Australia; f Cogstate Ltd., Melbourne, Victoria, Australia

**Keywords:** Risk factors, Cognition, Neuropsychology, Dementia risk, Greenspace, Social determinants of health, Neighborhood built environment

## Abstract

Dementia disproportionately affects individuals from disadvantaged backgrounds, including those living in areas of lower neighborhood-level socioeconomic status. It is important to understand whether there are specific neighborhood characteristics associated with dementia risk factors and cognition which may inform dementia risk reduction interventions. We sought to examine whether greenspace, walkability, and crime associated with the cumulative burden of modifiable dementia risk factors and cognition. This was a cross-sectional analysis of 2016–2020 data from the Healthy Brain Project, a population-based cohort of community-dwelling individuals across Australia. Participants were aged 40–70 and free of dementia. Measures included greenspace (greenspace % in the local area, and distance to greenspace, n = 2,181); and intersection density (n = 1,159), and crime (rate of recorded offences; n = 1,159). Outcomes included a modified Cardiovascular Risk Factors, Aging, and Incidence of Dementia (CAIDE) dementia risk score to index the burden of modifiable vascular dementia risk factors; and composite scores of both memory and attention, derived from the Cogstate Brief Battery. Linear regressions adjusted for age, sex, education, and personal socio-economic status, demonstrated distance to greenspace (*b* ± *SE* per 2-fold increase = 0.09 ± 0.03, *p* =.005) and crime rate (*b* ± *SE* per 2-fold increase = 0.07 ± 0.03, *p* =.018) were associated with higher modified CAIDE. Higher crime was associated with lower memory performance (*b* ± *SE* = -0.03 ± 0.01, *p* =.018). The association between distance to greenspace and modified CAIDE was only present in low-moderate socioeconomic status neighborhoods (p interaction = 0.004). Dementia prevention programs that address modifiable risk factors in midlife should consider the possible role of neighborhood characteristics.

## 1 Introduction

Dementia disproportionately affects the disadvantaged, including those living in areas of lower neighborhood-level socioeconomic status (n-SES) (Welberry et al., 2020). We recently observed that individuals residing in lower n-SES areas had higher dementia risk scores (i.e., higher Cardiovascular Risk Factors, Aging and Dementia [CAIDE] dementia risk scores) and poorer memory performance, even in midlife (Pase et al., 2022). Such findings provide a basis for investigating the specific neighborhood characteristics associated with dementia risk and cognition. Factors identified could inform targeted dementia risk reduction interventions aimed at reducing disparities in dementia and its risk factors. Possible candidates include features of the neighborhood built environment, such as access to greenspace and walkability, and characteristics of the neighborhood social environment, such as crime (Chen et al., 2022, Besser, 2021, Jimenez et al., 2022, Ng et al., 2018, Lee and Waite, 2018).

Beneficial effects of greenspace have been observed on physical and mental health and are theorised to occur through three interrelated pathways: 1) Mitigation: reducing harm, for example from environmental stressors such as air pollution, noise and heat; 2) Restoration: restoring the capacity of psychophysiological processes such as improved attention and reduced stress; and 3) Instoration: building the capacity for physical activity and social cohesion (Markevych et al., 2017). Several studies suggest that residing in areas with greater greenspace may promote optimal cognitive and brain health. However, such findings are equivocal, (Liu et al., 2020, Astell-Burt et al., 2020, Slawsky et al., 2022, Yuchi et al., 2020, Brown et al., 2018, Wu et al., 2020, Godina et al., 2023) with previous reviews noting substantial heterogeneity (Besser, 2021, de Keijzer et al., 2016, de Keijzer et al., 2020). Previous studies were limited by small sample sizes, (Cherrie et al., 2018, Watts et al., 2015) use of cognitive screening measures rather than individual cognitive domains (Koohsari et al., 2019) examination of cognition in older age alone rather than midlife (Brown et al., 2018) (where there might be more time for interventions to be implemented prior to dementia), and examining relatively restricted geographically areas (Yuchi et al., 2020, Brown et al., 2018, Cherrie et al., 2018). Finally, most previous studies have defined greenspace as “green” coverage using satellite imagery which indicates overall “greenness” of an area, such as the Normalized Difference Vegetation Index (NDVI). While this may link to cognitive health through psychophysiologically restorative processes that reduce stress (Markevych et al., 2017), it doesn’t necessarily capture access to greenspace (e.g., amount or proximity to parkland), which may associate with brain and cognitive health via other mechanisms, such as its relationship with lifestyle behaviors, including exercising or socialising (Besser, 2021). Investigation of cognition and greenspace as indexed by parkland has been relatively understudied, but as physical activity and cardiometabolic health are considered modifiable risk factors for dementia (Livingston et al., 2020), further exploration of access to parkland will be an important form of greenspace to examine in relation to cognition (Besser, 2021). Moreover, few studies have examined neighborhood walkability’s association with brain and cognitive health (Chen et al., 2022), which may be associated through physical activity and social connectedness.

In addition to the built environment, aspects of the neighbourhood social environment, such as crime, may also contribute to disparities in dementia risk. Crime, fear of crime, and neighborhood disorder are associated with poorer physical and psychological health, including psychological distress and social isolation (Ross and Mirowsky, 2009), and can negatively impact people’s use of public space, socialising, and going out (Robinson and Keithley, 2000, Lorenc et al., 2012). Very high rates of violent crime were associated with poorer cognition on an objective screening tool (Lee and Waite, 2018). However, there have been limited studies examining associations between crime and cognition in aging (Chen et al., 2022).

The primary aim of the current study was to determine whether objective neighborhood characteristics, particularly greenspace, walkability, and crime were associated with the cumulative burden of modifiable vascular dementia risk factors (summed together in a dementia risk factor score), and objective measures of cognition in an Australian community-sample aged 40 to 70. We hypothesised that higher greenspace access, higher walkability, and lower rates of crime would be associated with lower dementia risk profiles comprising vascular risk factors, and superior cognitive performance. Due to previous findings demonstrating that unfavorable neighborhood characteristics are more strongly associated with negative brain health outcomes in those with low socio-economic status (SES) (Cherrie et al., 2018), an exploratory aim was to determine whether any such relationships were moderated by n-SES, personal SES, or residential location (urban vs. rural geographical area).

## 2 Methods

### 2.1 Participants

The HBP is a prospective, community-based cohort of predominantly middle-aged Australians. As data collection occurs online, participants are located Australia-wide, including in regional and remote areas. As described previously (Lim et al., 2019), participants were aged 40–70 years at baseline, fluent in English, and free of self-reported significant neurological or psychiatric conditions, including dementia, or use of Alzheimer’s disease medications. The current study examined cross-sectional data from participants who undertook baseline assessment between 2016 and 2020. This research was approved by the Monash University Human Research Ethics Committee (#26855), and all participants provided written informed consent.

### 2.2 Measures

**Fig. 1:**
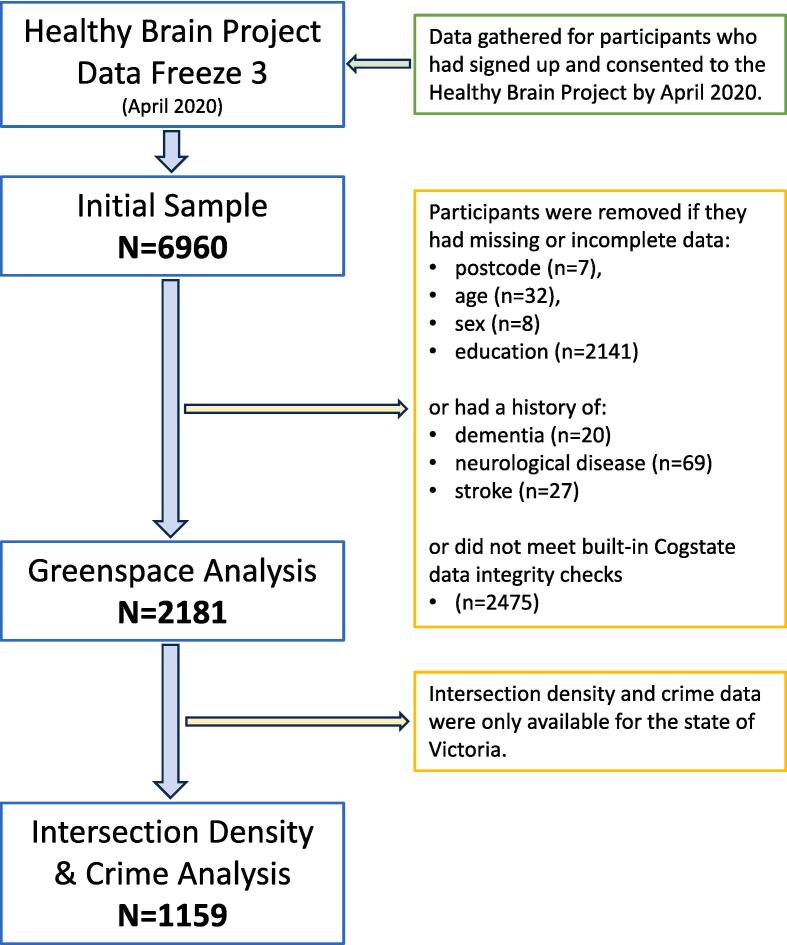
Participant flow diagram of Healthy Brain Project participants included in analyses.

The following neighborhood measures were publicly available and linked to this existing well-characterised cohort through postcodes (i.e., zipcode). For measures of greenspace, Australia-wide data were used (N = 2,181), while for measures of crime and intersection density, only participants residing in the state of Victoria were included (N = 1,159). This was because data on crime were available at the postcode level for Victoria only and because there are no nationally consistent data for intersection density, but detailed data are available for Victoria, in which most of the study sample (53 %) reside (see Fig. 1).

#### 2.2.1 Greenspace

**Fig. 2:**
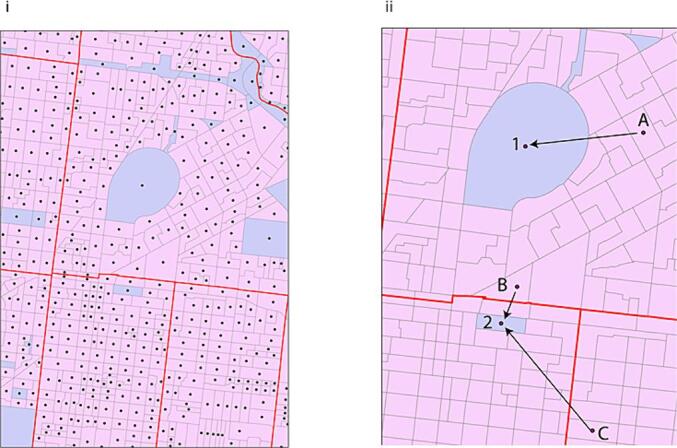
Example of how geographical meshblocks are used to index greenspace **i**. A snapshot of Melbourne captured from ArcMap 10.8. Red borders indicate postcode boundaries, the smaller subsections are meshblocks (the smallest unit of geographic area, as defined by ABS [2016]), and purple meshblocks indicate parkland/greenspace. Geometric centroid points are derived for each meshblock and are used as the reference point for the calculation of distance, as indicated by the black dots. **ii.** Distance from each meshblock centroid to the nearest greenspace meshblock is calculated in metres. For example, *meshblock A* is closest to *greenspace 1*, *meshblock B* is closest to *greenspace 2* despite being in a different postcode, and *meshblock C* is closest to *greenspace 2* despite being some distance away. *Meshblock C* would have a higher distance to greenspace score than that of *meshblock A* or *B*. This distance score is calculated for each meshblock, then weighted-meshblock scores (by population; higher population = greater weighting) are averaged to create a single index score for each postcode. Each participant is assigned the indexed score according to their postcode to be used in analyses. (For interpretation of the references to colour in this figure legend, the reader is referred to the web version of this article.)

Greenspace was indexed by i) the percentage of greenspace within a postcode, and ii) the average population-weighted distance to greenspace for each postcode. Greenspace was defined as parkland as identified from MeshBlocks, the smallest unit of population-level geographic area, based on Australian Bureau of Statistics (ABS) 2016 census population data (Australian Bureau of Statistics, 2016). Percentage greenspace for each postcode area was calculated. Additionally, the average distance to the nearest greenspace for each postcode was determined by converting each MeshBlock to a centroid point, the geometric middle of the area. The distance between that point and the nearest parkland MeshBlock was calculated for each MeshBlock. The total distance to the nearest parkland was calculated per MeshBlock, with the total distance of all population to parkland collated, then divided by population to give a population-weighted average distance to parkland. To obtain a postcode-level index for both percentage greenspace and distance to greenspace, MeshBlocks within each postcode were averaged (Fig. 2).

#### 2.2.2 Crime

Crime data were obtained from the Crime Statistics Agency based on data provided by the ABS (Crime Statistics Agency, 2021). The rate of recorded offences was calculated per 100,000 population from July 2011 to June 2021. All types of offences were included.

#### 2.2.3 Street Intersection density

Street intersection density per square kilometre was used to index walkability. The number of street intersections for each postcode in Victoria was calculated using data from the Victorian State Government open data portal, (Department of Environment Water and Planning, 2021) and analysed using ArcMap 10.8 geospatial software. Street intersection density was then calculated by dividing the number of intersections per postcode by the number of square kilometres per postcode, with higher street intersection density reflecting higher walkability.

#### 2.2.4 CAIDE dementia risk score

The CAIDE Dementia Risk Score (Kivipelto et al., 2006), is computed based on demographics (i.e., age, sex, and education) and vascular risk factors (i.e., hypertension, body mass index [BMI], hypercholesterinemia, physical activity), with a higher score indicating higher risk (see eTable 1 for details). It has shown good predictive accuracy for the subsequent development of late-life dementia (Kivipelto et al., 2006). As we have done previously, we used a modified CAIDE dementia risk score for the current study by removing age, sex, and education to determine whether any found relationships are independent of factors that are unmodifiable (i.e., age and sex) or not readily modifiable (i.e., education). We kept the CAIDE score in its original units, as is standard and which can be compared across studies. We used the modified CAIDE as our primary outcome given our interest in examining whether neighbourhood characteristics are associated with the portion of dementia risk that is readily modifiable. Moreover, our sample was predominantly middle-aged and the vascular risk factors that comprise the modified CAIDE exert their strongest impact on late-life dementia risk when present from midlife.

#### 2.2.5 Cognition

To assess cognition remotely and unsupervised, the Cogstate Brief Battery (CBB), an online cognitive assessment platform was used, as described previously (Lim et al., 2012). The validity of the unsupervised format has been demonstrated in healthy middle-aged and older adults with comparable accuracy to the supervised format, and decrements in accuracy and speed with both increasing task complexity and age (Perin et al., 2020). An attention composite was calculated from the Detection and Identification tasks, while a memory composite was calculated from the One Card Learning and One-Back tasks, from z-scores on the individual tests. We derived z-scores because the units are arbitrary and this is the most common approach for handling these outcomes. Higher scores indicated better performance (see eMethods for further details).

#### 2.2.6 Socio-economic status

N-SES was categorised into the highest (≥8-10th deciles) versus lower (<8th decile) as per our previous approach (Pase et al., 2022) using the ABS Index of Relative Socioeconomic Advantage and Disadvantage (IRSAD). This index ranks Australian neighborhoods from least to most advantaged based on socioeconomic characteristics of individuals and households within postcodes. Personal SES was coded using the Australian Socioeconomic Index 2006 based on occupation. Higher scores represented higher personal SES (McMillan et al., 2009) (see eMethods for further details).

#### 2.2.7 Geographical location

Geographical location was categorized as urban (i.e., major city) or non-urban (inner regional, outer regional, remote, very remote) based on the Accessibility/Remoteness Index of Australia categories, which categorizes locations by measures of road distance to the nearest service centers based on population (Hugo Centre for Population and Migration Studies, 2021).

### 2.3 Data analysis

Analyses were conducted in SPSS, version 28 and R version 4.0.3. Large outliers on crime (median ± interquartile range x 3) were winsorized at 1 unit outside the next most extreme score in the distribution. For descriptive purposes, Pearson correlations were estimated between all neighborhood predictors, and moderators. A log base 2 transformation was applied to greenspace area, distance to greenspace, and crime to better reflect an expected non-linear (i.e., asymptotic) relationship with the outcomes.

#### 2.3.1 Primary analyses

Separate multivariable linear regressions were fitted to estimate the association between each neighborhood characteristic and modified CAIDE dementia risk scores or cognition. Covariates included age, sex, education categories (equivalent to high school or less, undergraduate degree, and postgraduate study), and personal SES. Unstandardised beta (b) regression weights and standard errors (SE) are reported for regression models. Regression weights for log2 transformed variables represent the mean change in the outcome for a 2-fold increase in the predictor. In addition to separate multivariable linear regression models, a model was fitted that included all neighborhood characteristics together. This allowed for the examination of the independent association for each neighborhood predictor (i.e., the “effect” of changing a given neighborhood characteristic while all others are held constant).

As the modified CAIDE and cognitive composite scores do not have intrinsically meaningful units, the magnitude of effect sizes (regression weights) for these outcomes can be difficult to interpret. Consequently, to provide some benchmark for their interpretation, we present the number of years of age that would be required to produce, on average, an equivalent change in the outcome (estimated by unadjusted linear regression).

#### 2.3.2 Exploratory analyses

Moderation analyses examined whether neighborhood characteristics interacted with n-SES (1st-7th decile, 8th-10th decile), personal SES (continuous), or geographical location (urban, non-urban) on the outcomes in a multivariable model including each interaction term. Where evidence of moderation was noted (*p* interaction < 0.05), results are presented within strata of the modifying variable. Moderation models were adjusted for age, sex, education, and personal SES. The threshold for significance within-strata was a *p*-value of < 0.05.

## 3 Results

### 3.1 Sample characteristics

Approximately three quarters of the sample resided in urban areas (72% and 77% in Australia and Victoria, respectively). The average age was 56 years, most were female (76% and 73% for Australia and Victoria, respectively) and the average years of education completed was 16 (see Table 1). Relationships were observed between and amongst several neighborhood-level factors and SES moderators (see eTable 2).

**Table 1:** Baseline demographic characteristics of Healthy Brain Project participants – Australia-wide and Victorian-only samples, 2016–2020.

	*Australia-wide sample (n = 2,181)*		*Victorian-only sample (n = 1,159)*	
	*M (SD)/Mdn(IQR)*	*Range*	*M (SD)/Mdn(IQR)*	*Range*
Age	56.75 (7.00)	40.03–70.98	56.50 (6.83)	40.03–70.98
Sex (female) n (%)	1,652 (75.7)		849 (73.3)	
Education (years)	15.97 (3.45)	5–24	16.01 (3.46)	6–24
High school or less n (%)	361 (16.6)		193 (16.7)	
Undergraduate degree n (%)	539 (24.7)		287 (24.8)	
Postgraduate degree n (%)	1,281 (58.7)		679 (58.6)	
n-SES	7.26 (2.91)	1–10	7.53 (2.81)	1–10
Personal SES	66.63 (19.17)	11–100	66.79 (19.02)	17.1–100
Residing in an urban area (%)	1,578 (72.4)		902 (77.8)	
Greenspace % area^	14.40 (17.91)	0–95.36	11.54 (14.37)	0–95.36
Distance to greenspace distance m^	263.18 (194.41)	85.11–21,953	279.22 (166.99)	85.12–16,231.43
Street intersection density per km^2^^	55.24 (51.80)	<0.01–196.13	57.29 (47.83)	0.44–196.13
Crime rate per 100,000 population^	1,515 (,1872.00)	2–19,576	1,572.00 (1,872.00)	2–19,576
CAIDE	4.99 (2.26)	0–13	5.10 (2.29)	0–13
mCAIDE	1.35 (1.64)	0–7	1.42 (1.67)	0–7
Memory	-0.0003 (0.77)	−3.54–2.57	0.0206 (0.80)	−3.54–2.57
Attention	0.0003 (0.90)	−5.50–2.59	0.0057 (0.88)	−4.50–2.44

^Median and Interquartile range provided for skewed variables; n-SES = neighborhood-level socio-economic status; SES = socio-economic status; CAIDE = Cardiovascular Risk Factors, Aging, and Incidence of Dementia; mCAIDE = modified Cardiovascular Risk Factors, Aging, and Incidence of Dementia.

### 3.2 Associations between neighborhood characteristics and CAIDE dementia risk scores

After controlling for age, sex, education and personal SES, each 2-fold increase in distance to greenspace was associated with a mean increase of 0.09 units in the modified CAIDE score (±*SE* = 0.03, *p* =.005); and each 2-fold increase in crime rate was associated with a mean increase of 0.07 units in the modified CAIDE score (±*SE* = 0.03, *p* =.018). To contextualise effect sizes, this was approximately equivalent to the average increase in modified CAIDE score attributable to a 2.5- and 2-year increase in age, respectively. There were no significant associations between the percentage area of greenspace or intersection density with the modified CAIDE dementia risk score (Table 2). Estimates were similar in the mutually-adjusted model including all neighborhood characteristics, though standard errors tended to be larger and only crime was significantly associated with modified CAIDE score (*b* ± *SE* = 0.14 ± 0.03, *p* < 0.001) (Table 2).

**Table 2:** Cross-sectional associations between dementia risk scores and cognition by neighbourhood characteristics in the Healthy Brain Project sample, 2016–2020.

	mCAIDE		Memory		Attention	
	*b (SE)*	*p*	*b (SE)*	*p*	*b (SE)*	*p*
*Independent models*						
Log2 % Greenspace area	-0.03(0.02)	0.164	-0.01(0.01)	0.200	0.00(0.01)	0.883
Log2 Greenspace distance	**0.09 (0.03)**	**0.005**	0.00 (0.02)	0.953	0.00(0.02)	0.919
Intersection density, per 50 per km^2^	-0.12(0.06)	0.067	0.02(0.03)	0.508	0.02(0.03)	0.491
Log2 crime rate^O^	**0.07(0.03)**	**0.018**	**-0.03(0.01)**	**0.018**	-0.02(0.02)	0.234
*Mutually adjusted model*						
Log2 % Greenspace area	-0.02(0.03)	0.632	-0.01(0.02)	0.336	-0.02(0.02)	0.329
Log2 Greenspace distance	0.12(0.07)	0.116	-0.01(0.04)	0.879	-0.03(0.04)	0.452
Intersection density, per 50 per km^2^	-0.16(0.08)	0.064	0.06(0.04)	0.181	-0.03(0.04)	0.490
Log2 crime rate^O^	**0.14(0.03)**	**<0.001**	**-0.04(0.02)**	**0.012**	-0.02(0.02)	0.349

mCAIDE = modified Cardiovascular Risk Factors, Aging and Dementia; IRSAD = Index of Relative Socio-economic Advantage and Disadvantage; SES = socio-economic status. Age, sex, education, and personal SES were used as covariates in models. Winsorized variables are denoted with superscript “O”.

### 3.3 Associations between neighborhood characteristics and memory and attention

Each 2-fold increase in crime was associated with a mean decrease of 0.03 units in memory composite score (*SE* = 0.1, *p* =.018). This was approximately equivalent to the reduction in memory score attributable to a 3-year increase in age. No significant associations were observed between measures of greenspace or intersection density and memory, controlling for age, sex, education, and personal SES. In the mutually adjusted model including all neighborhood characteristics, only crime was significantly associated with memory score (*b* ± *SE* = -0.04 ± 0.02, *p* =.012). None of the exposures were significantly associated with attention (Table 2).

### 3.4 Moderators of neighborhood characteristics and CAIDE dementia risk score and cognition

There was a significant interaction between distance to greenspace and n-SES on the modified CAIDE (*p* interaction = .004). For those residing in areas of lower n-SES, a 2-fold increase in distance to greenspace was associated with a mean increase of 0.20 units on the modified CAIDE (*SE* = 0.07, *p* =.004), while there was no evidence of an association for those residing in the areas of highest n-SES (*b* ± *SE* = –0.01 ± 0.06, p =.852). No other significant interactions were observed (Table 3).

**Table 3:** Summary of moderation analyses of cross-sectional associations between neighborhood characteristics and dementia risk scores or cognition in the Healthy Brain Project sample, 2016–2020.

			***Interaction p value*^a^ for each outcome**		
**Exposure**	*Moderator*	*Stratification*	**m-CAIDE**	**Memory**	**Attention**
**% Area Greenspace**	*n-SES*		0.394	0.458	0.517
	*Personal SES*		0.464	0.476	0.383
	*Location*		0.504	0.659	0.747
**Distance to greenspace**	*n-SES*		**0.004**	0.838	0.880
		Lower n-SES	*b(SE) =* **0.20(0.07), *p* =.004**		
		Highest n-SES	*b(SE) =* -0.01(0.06), *p* =.852		
	*Personal SES*		0.574	0.326	0.307
	*Location*		0.805	0.800	0.917
**Intersection** density	*n-SES*		0.982	0.844	0.935
	*Personal SES*		0.852	0.847	0.079
	*Location*		0.270	0.282	0.892
**Crime**	*n-SES*		0.371	0.675	0.386
	*Personal-SES*		0.688	0.566	0.719
	*Location*		0.594	0.304	0.560

n-SES = neighborhood-level socio-economic status; SES = socio-economic status; mCAIDE = modified Cardiovascular Risk Factors, Aging, and Incidence of Dementia. ^a^Interaction p value reported unless specified otherwise. Results are stratified in the presence of a significant interaction effect (p <.05). Location refers to geographical location (i.e., urban vs. non-urban).

## 4 Discussion

This study examined whether neighborhood characteristics were associated with the cumulative burden of modifiable vascular dementia risk factors or cognition in a community sample. Shorter distance to greenspace and lower crime were each associated with a lower burden of modifiable vascular dementia risk factors. Lower crime was associated with superior memory performance, in line with expectations, whereas intersection density and greenspace were not. Neighborhood characteristics were not associated with attention performance.

Longer distance to greenspace was associated with higher modifiable dementia risk scores. These findings are broadly consistent with previous literature demonstrating an association between more residential greenspace and better health (Sallis et al., 2020). However, this association did not extend to cognition, specifically memory and attention, which contrasts with some prior research, (Jimenez et al., 2022, Cherrie et al., 2018, de Keijzer et al., 2018) including a previous study where the effect was observed for attention (Jimenez et al., 2022). One possible explanation for the difference between studies is the way greenspace was defined, with the current study using parkland, while others have used the NDVI which identifies vegetation quantity based on pixels of satellite images; or forest greenspace/canopy cover based on satellite images (Astell-Burt et al., 2020, Godina et al., 2023). Better access to parks associates with higher levels of physical activity (Sallis et al., 2020) which in turn can reduce vascular risk factors (hence explaining the current observation with the modified CAIDE), while overall “greenness” around the home may improve attention through psychophysiological stress recovery (Markevych et al., 2017). Alternatively, associations with cognition may take longer to become apparent over time, compared to associations with vascular risk factors. This is a limitation of the cross-sectional study design. A direct comparison of NDVI and levels of forest or tree canopy cover compared to greenspace access would be worthwhile in future research.

There was no association found between percentage of greenspace area and CAIDE scores or cognition, consistent with prior research indicating a lack of findings with overall greenspace within a given area and cognitive and brain health outcomes (Yuchi et al., 2020, Wu et al., 2020, Godina et al., 2023). This may have potential implications for public policy, suggesting that better access (i.e., closer proximity) to parkland may be more important than the amount of public parkland for dementia risk reduction. In other words, having more smaller parks in urban centres may be more beneficial to health than fewer larger parks. This is consistent with international research demonstrating an association between closer perceived park proximity and lower BMI, overweight and obesity (Sallis et al., 2020), however, further research is required to explore this possibility. While we expected different associations in urban areas (where greenspace may link to lifestyle behaviors) compared to rural areas (where parkland may be less relevant to lifestyle) this was not borne out in the data and requires further consideration.

Higher intersection density was not associated with lower dementia risk scores. A previous Australian study found that *better* street connectivity was associated with *higher* risk of dementia diagnosis, possibly due to older individuals more likely to receive a dementia diagnosis, living in older suburbs with better street connectivity (Bagheri et al., 2021). This is particularly relevant to the Australian context, where significant differences in walkability across suburbs and cities can depend on the period in which the suburb was established, and hence the demographic makeup of residents. For example, the garden city movement in early twentieth century planning, promoted inclusion of large public parks, while many suburbs developed later were planned without adequate provision for public open space (Howe and Howe, 1988). Additionally, in Bagheri et al. (Bagheri et al., 2021), walkability was not associated with dementia risk score. In both that study and the current, walkability was calculated at the area (SA1) level, while previous studies showing associations between better walkability and positive health outcomes measured walkability in area buffers around each participant’s address (Sallis et al., 2020, Frank et al., 2006), although not always (McCormack et al., 2020). Therefore, walkability measured within a residential buffer may be a more important determinant of health (through actual walking, the opportunity for spontaneous social interaction, or reduced air pollution), rather than the general level of walkability of the entire suburb which, in Australia, can cover vast areas.

Higher crime was associated with higher modifiable dementia risk factor scores and lower memory performance. These associations may be due to behavioural changes in response to higher crime rates and any associated stress, such as reducing outdoor and social activity, and sleep, or increased alcohol use and smoking (Franks et al., 2021, Foster and Giles-Corti, 2008). This explanation is supported by research demonstrating that social disorder associates with psychological distress and mistrust (Ross and Mirowsky, 2009). Alternatively, people with lower education and poorer health, may have less financial capacity to choose to live in more desirable neighborhoods with lower levels of crime, although the association between crime and memory performance remained after controlling for education and personal SES.

The association between distance to greenspace and modifiable vascular dementia risk factors were observed only for individuals residing in areas of low to intermediate n-SES and not the highest level of n-SES, consistent with previous findings demonstrating stronger effects between neighborhood characteristics and health or cognition for those with lower SES (Cherrie et al., 2018, Rodrigues et al., 2021). The weathering hypothesis posits that structural disadvantage results in multiple chronic stressors and requires sustained effort to cope, placing higher physiological demand on individuals that ultimately leads to advanced biological ageing (Geronimus et al., 2006, Simons et al., 2021). In this context, the results may reflect that living in areas with limited access to parkland is a stressor that disproportionately affects those who may already be vulnerable on the basis of living in an area of lower SES, and those with lower SES having less capacity to take advantage of spaces beyond their immediate environment (Cherrie et al., 2018). However, we cannot rule out the possibility of residual confounding in evaluating relationships in this study.

In comparison to models in which individual neighborhood characteristics were examined separately, in a mutually adjusted model holding all other neighborhood characteristics constant, only higher crime remained clearly associated with higher modified CAIDE and lower memory. Nevertheless, in the case of the modified CAIDE, the estimated association for distance to greenspace was slightly larger in this mutually adjusted model than the independent model. The non-significance of this association is therefore likely attributable to the reduced sample size available for the mutually adjusted model, which was restricted to data only from Victoria. As crime has been relatively understudied with respect to cognition and dementia risk factor burden, the results highlight a need to further explore this feature of the neighborhood environment.

There are several strengths of this research. We leveraged a large, well-characterised, geographically dispersed, and predominantly middle-aged cohort and linked this to objective measures of the neighborhood-built environment, and official records of the social environment, rather than relying on subjective perceptions of place, a limitation of previous research. There are also several limitations to the current study. The measure of greenspace as defined by the ABS was broad, for example private golf courses and sporting fields are counted the same as public parks and recreation zones. Furthermore, given our neighborhood measures are at the level of postcode rather than residential address, there is some measurement error regarding the exposures of interest, and activity data is not available to verify actual behaviors. Our use of official crime rates does not capture personal victimization of individuals within the study. However, as a relatively low incidence of crime was observed overall, it is not believed this would drive the findings, as noted by others (Ross and Mirowsky, 2009). Other features of neighborhoods not captured but that may also be important include air pollution, given direct (neurotoxic) and indirect (cerebrovascular) links with dementia (Peters et al., 2019), noise pollution, and heat vulnerability as markers of environmental socio-economic disadvantage (Tanton et al., 2021) which may also impact physical activity. There may also be important differences in findings depending on regional climate and cultural attitudes towards the natural environment (Besser, 2021). The sample included here is English-speaking, relatively educated and socioeconomically advantaged compared to the general population, making it difficult to fully explore interactions with low SES, and limiting generalizability of the results to the general Australian population. Relatedly, participants were overall healthy, with the average modified CAIDE score indicating low levels of modifiable vascular risk in the sample. This limited the degree to which relationships could be examined in individuals with higher vascular dementia risk factor burden. While the CAIDE is a good measure of modifiable *vascular* dementia risk factor burden, examination of other modifiable dementia risk factors, such as social and cognitive engagement, diet, and sleep in relation to neighborhood characteristics would also be beneficial, for example using a risk factors scale such as the CogDrisk (Anstey et al., 2022). Finally, we used participants’ current postcode, and therefore it cannot be guaranteed that neighborhood characteristics temporally precede outcomes.

This study demonstrates that closer proximity to greenspace, and lower crime rates are associated with the burden of modifiable vascular dementia risk factors with potential modifying effects of n-SES; and that lower crime is associated with better memory performance. Future research is required to understand these relationships more thoroughly (i.e., through different neighborhood measures and in more diverse populations) over time, with measurement of length of time lived within a given area. Greater efforts to include more diversity of participants in research studies, e.g., with respect to individual or area-level SES, educational attainment, and digital or health literacy will be important for growing the body of evidence that aims to understand mechanisms through which various measures of disadvantage are associated with poorer health and increased dementia risk. Examination of other outcomes, such as executive function, dementia, and brain MRI outcomes in prospective studies would also be informative. Although research linking neighbourhood characteristics and dementia risk is still in its infancy, associations with some health outcomes are well established. Therefore, dementia prevention programs that seek to improve modifiable dementia risk factors through improving healthy behaviours should consider the potential influence of neighborhood characteristics on intervention adherence. For example, an area with less access to greenspace, and higher crime rates may pose barriers to participation in or adherence to an exercise or social engagement program. Such barriers may unintentionally bias studies to individuals who live in more advantaged areas, further excluding those who are disadvantaged and who would benefit the most from interventions designed to reduce dementia risk. Policy interventions by different levels of government could address social determinants of health at the neighborhood level, potentially improving health and thereby reducing dementia risk. This approach is now gaining traction in mental illness prevention (Patton et al., 2021, Lund et al., 2018), where improving neighborhood safety was proposed as an intervention that could result in substantial positive outcomes (Lund et al., 2018). Additionally, the World Health Organization argues that collaboration between health and non-health sectors (e.g., environment, infrastructure, housing) is required to harmonize resources and improve equity in relation to dementia policy making (World Health Organization, 2018). Dementia *risk reduction* policies may also benefit from this approach to improve the sustainability and scalability of prevention approaches.

## Funding

The Healthy Brain Project is funded by grants AARG-17-591424, AARG-18-591358, and AARG-19-643133 from the Alzheimer’s Association; grants GNT1158384, GNT1147465, GNT1111603, GNT1105576, GNT1104273, GNT1158384, and GNT1171816 from the NHMRC of Australia; and funding from the Bethlehem Griffiths Research Foundation, the Dementia Australia Research Foundation, and the Yulgilbar Alzheimer’s Research Program. YY Lim is supported by an NHMRC Career Development Fellowship (GNT1162645) and an NHMRC Emerging Leadership Grant (GNT2009550). MP is funded by an NHMRC Emerging Leadership Grant (GTN2009264).

## CRediT authorship contribution statement

**Marina G. Cavuoto:** Writing – review & editing, Writing – original draft, Methodology, Formal analysis, Data curation, Conceptualization. **Liam Davies:** Writing – review & editing, Writing – original draft, Visualization, Methodology, Data curation, Conceptualization. **Ella Rowsthorn:** Writing – original draft, Visualization, Project administration, Methodology, Data curation. **Lachlan G. Cribb:** Writing – review & editing, Writing – original draft, Methodology, Formal analysis. **Stephanie R. Yiallourou:** Writing – review & editing, Writing – original draft, Methodology. **Nawaf Yassi:** Writing – review & editing, Funding acquisition. **Paul Maruff:** Writing – review & editing, Resources, Funding acquisition. **Yen Ying Lim:** Writing – review & editing, Resources, Funding acquisition. **Matthew P. Pase:** Writing – review & editing, Writing – original draft, Supervision, Resources, Methodology, Funding acquisition, Data curation, Conceptualization.

## Declaration of competing interest

The authors declare the following financial interests/personal relationships which may be considered as potential competing interests: Dr Cavuoto and Dr Pase are supported by a Dementia Australia Research Foundation award (Lucas' Papaw Remedies Project Grant). Dr Pase reported receiving grants from the Alzheimer’s Association, the Bethlehem Griffiths Research Foundation, the National Health and Medical Research Council (NHMRC), and the National Heart Foundation of Australia during the conduct of the study and grants from the Alzheimer's Disease Drug Discovery Foundation, the Brain Foundation, the National Institutes of Health, the NHMRC, and the Stroke Foundation outside the submitted work. Dr Lim reported receiving grants from the NHMRC outside the submitted work. Dr Maruff is a full-time employee of Cogstate Ltd, the company that provided the Cogstate Brief Battery. No other disclosures were reported.

## Data Availability

Data will be made available on request.

## References

[b0005] Anstey K.J., Kootar S., Huque M.H., Eramudugolla R., Peters R. (2022). Development of the CogDrisk tool to assess risk factors for dementia. Alzheimer's & Dementia: Diagnosis, Assessment & Disease Monitoring.

[b0010] Astell-Burt T., Navakatikyan M.A., Feng X. (2020). Urban green space, tree canopy and 11-year risk of dementia in a cohort of 109,688 Australians. Environ. Int..

[b0015] Australian Bureau of Statistics. 1270.0.55.001 - Australian Statistical Geography Standard (ASGS): Volume 1 - Main Structure and Greater Capital City Statistical Areas [online]. Available at: https://www.abs.gov.au/AUSSTATS/abs@.nsf/DetailsPage/1270.0.55.001July%202016?OpenDocument.

[b0020] Bagheri N., Mavoa S., Tabatabaei-Jafari H. (2021). The Impact of Built and Social Environmental Characteristics on Diagnosed and Estimated Future Risk of Dementia. J. Alzheimers Dis..

[b0025] Besser L. (2021). Outdoor green space exposure and brain health measures related to Alzheimer’s disease: a rapid review. BMJ Open.

[b0030] Brown S.C., Perrino T., Lombard J. (2018). Health disparities in the relationship of neighborhood greenness to mental health outcomes in 249,405 US Medicare beneficiaries. Int. J. Environ. Res. Public Health.

[b0035] Chen X., Lee C., Huang H. (2022). Neighborhood built environment associated with cognition and dementia risk among older adults: A systematic literature review. Soc. Sci. Med..

[b0040] Cherrie M.P., Shortt N.K., Mitchell R.J. (2018). Green space and cognitive ageing: A retrospective life course analysis in the Lothian Birth Cohort 1936. Soc Sci Med.

[b0045] Crime Statistics Agency. Latest Victorian crime data [online]. Available at: https://www.crimestatistics.vic.gov.au/crime-statistics/latest-victorian-crime-data/download-data. Accessed 30th November, 2021.

[b0050] de Keijzer C., Gascon M., Nieuwenhuijsen M.J., Dadvand P. (2016). Long-term green space exposure and cognition across the life course: a systematic review. Current Environ. Health Reports.

[b0055] de Keijzer C., Tonne C., Basagaña X. (2018). Residential surrounding greenness and cognitive decline: a 10-year follow-up of the Whitehall II cohort. Environ. Health Perspect..

[b0060] de Keijzer C., Bauwelinck M., Dadvand P. (2020). Long-term exposure to residential greenspace and healthy ageing: A systematic review. Current Environ. Health Reports.

[b0065] Department of Environment L, Water & Planning,. Road Infrastructure - Vicmap Transport [online]. Available at: https://discover.data.vic.gov.au/dataset/road-infrastructure-vicmap-transport. Accessed 2nd December, 2021.

[b0070] Foster S., Giles-Corti B. (2008). The built environment, neighborhood crime and constrained physical activity: an exploration of inconsistent findings. Prev. Med..

[b0075] Frank L.D., Sallis J.F., Conway T.L., Chapman J.E., Saelens B.E., Bachman W. (2006). Many pathways from land use to health: associations between neighborhood walkability and active transportation, body mass index, and air quality. J. Am. Plann. Assoc..

[b0080] Franks K.H., Bransby L., Saling M.M., Pase M.P. (2021). Association of stress with risk of dementia and Mild Cognitive Impairment: a systematic review and meta-analysis. J. Alzheimers Dis..

[b0085] Geronimus A.T., Hicken M., Keene D., Bound J. (2006). “Weathering” and age patterns of allostatic load scores among Blacks and Whites in the United States. Am. J. Public Health.

[b0090] Godina S.L., Rosso A.L., Hirsch J.A. (2023). Neighborhood greenspace and cognition: The cardiovascular health study. Health Place.

[b0095] Howe R., Howe R. (1988). New houses for old: fifty years of public housing in Victoria Ministry of Housing and Construction.

[b0100] Hugo Centre for Population and Migration Studies. Accessibility/Remoteness Index of Australia (ARIA). [online]. Available at: https://arts.adelaide.edu.au/hugocentre/services/aria. Accessed Updated June 22, 2021. Accessed June 4, 2020.

[b0105] Jimenez M.P., Elliott E.G., DeVille N.V. (2022). Residential Green Space and Cognitive Function in a Large Cohort of Middle-Aged Women. JAMA Netw. Open.

[b0110] Kivipelto M., Ngandu T., Laatikainen T., Winblad B., Soininen H., Tuomilehto J. (2006). Risk score for the prediction of dementia risk in 20 years among middle aged people: a longitudinal, population-based study. The Lancet Neurology.

[b0115] Koohsari M.J., Nakaya T., McCormack G.R. (2019). Cognitive function of elderly persons in Japanese neighborhoods: The role of street layout. Am. J. Alzheimers Dis. Other Demen..

[b0120] Lee H., Waite L.J. (2018). Cognition in context: The role of objective and subjective measures of neighborhood and household in cognitive functioning in later life. Gerontologist.

[b0125] Lim Y.Y., Ellis K.A., Harrington K. (2012). Use of the CogState Brief Battery in the assessment of Alzheimer's disease related cognitive impairment in the Australian Imaging, Biomarkers and Lifestyle (AIBL) study. J. Clin. Exp. Neuropsychol..

[b0130] Lim Y.Y., Yassi N., Bransby L., Properzi M., Buckley R. (2019). The Healthy Brain Project: an online platform for the recruitment, assessment, and monitoring of middle-aged adults at risk of developing Alzheimer’s disease. J. Alzheimers Dis..

[b0135] Liu C.-C., Sun Y., Kung S.-F. (2020). Effects of physical and social environments on the risk of dementia among Taiwanese older adults: a population-based case-control study. BMC Geriatr..

[b0140] Livingston G., Huntley J., Sommerlad A. (2020). Dementia prevention, intervention, and care: 2020 report of the *Lancet* Commission. Lancet.

[b0145] Lorenc T., Clayton S., Neary D. (2012). Crime, fear of crime, environment, and mental health and wellbeing: mapping review of theories and causal pathways. Health Place.

[b0150] Lund C., Brooke-Sumner C., Baingana F. (2018). Social determinants of mental disorders and the Sustainable Development Goals: a systematic review of reviews. Lancet Psychiatry.

[b0155] Markevych I., Schoierer J., Hartig T. (2017). Exploring pathways linking greenspace to health: Theoretical and methodological guidance. Environ. Res..

[b0160] McCormack G.R., Frehlich L., Blackstaffe A., Turin T.C., Doyle-Baker P.K. (2020). Active and fit communities. Associations between neighborhood walkability and health-related fitness in adults. Int. J. Environ. Res. Public Health.

[b0165] McMillan J., Beavis A., Jones F.L. (2009). The AUSEI06: A new socioeconomic index for Australia. J. Sociol..

[b0170] Ng T.P., Nyunt M.S.Z., Shuvo F.K. (2018). The neighborhood built environment and cognitive function of older persons: results from the Singapore longitudinal ageing study. Gerontology.

[b0175] Pase M.P., Rowsthorn E., Cavuoto M.G. (2022). Association of neighborhood-level socioeconomic measures with cognition and dementia risk in Australian dults. JAMA Netw. Open.

[b0180] Patton G.C., Raniti M., Reavley N. (2021). Rediscovering the mental health of populations. World Psychiatry.

[b0185] Perin S., Buckley R.F., Pase M.P. (2020). Unsupervised assessment of cognition in the Healthy Brain Project: Implications for web-based registries of individuals at risk for Alzheimer's disease. Alzheimer's & Dementia: Transl. Res. Clinical Interventions.

[b0190] Peters R., Ee N., Peters J., Booth A., Mudway I., Anstey K.J. (2019). Air pollution and dementia: a systematic review. J. Alzheimers Dis..

[b0195] Robinson F., Keithley J. (2000). The impacts of crime on health and health services: A literature review. Health Risk Soc..

[b0200] Rodrigues D.E., César C.C., Xavier C.C., Caiaffa W.T., Proietti F.A. (2021). Exploring neighborhood socioeconomic disparity in self-rated health: a multiple mediation analysis. Prev. Med..

[b0205] Ross C.E., Mirowsky J. (2009). Neighborhood disorder, subjective alienation, and distress. J. Health Soc. Behav..

[b0210] Sallis J.F., Cerin E., Kerr J. (2020). Built environment, physical activity, and obesity: findings from the International Physical Activity and Environment Network (IPEN) Adult Study. Annu. Rev. Public Health.

[b0215] Simons R.L., Lei M.-K., Klopack E., Beach S.R., Gibbons F.X., Philibert R.A. (2021). The effects of social adversity, discrimination, and health risk behaviors on the accelerated aging of African Americans: further support for the weathering hypothesis. Soc. Sci. Med..

[b0220] Slawsky E.D., Hajat A., Rhew I.C. (2022). Neighborhood greenspace exposure as a protective factor in dementia risk among US adults 75 years or older: a cohort study. Environ. Health.

[b0225] Tanton, R., Dare, L., Miranti, R., Vidyattama, Y., Yule, A., 2021. McCabe M. Dropping Off the Edge 2021: Persistent and multilayered disadvantage in Australia. Melbourne2021.

[b0230] Watts A., Ferdous F., Moore K.D., Burns J.M. (2015). Neighborhood integration and connectivity predict cognitive performance and decline. Gerontol. Geriatric Med..

[b0235] Welberry H.J., Brodaty H., Hsu B., Barbieri S., Jorm L.R. (2020). Measuring dementia incidence within a cohort of 267,153 older Australians using routinely collected linked administrative data. Sci. Rep..

[b0240] World Health Organization, 2018. Towards a dementia plan: a WHO guide. Geneva.

[b0245] Wu Y.-T., Brayne C., Liu Z. (2020). Neighbourhood environment and dementia in older people from high-, middle-and low-income countries: results from two population-based cohort studies. BMC Public Health.

[b0250] Yuchi W., Sbihi H., Davies H., Tamburic L., Brauer M. (2020). Road proximity, air pollution, noise, green space and neurologic disease incidence: a population-based cohort study. Environ. Health.

