# Supplementary material for: Cross-sectional associations between neighborhood characteristics, cognition and dementia risk factor burden in middle-aged and older Australians
Source: Prev Med Rep. 2024 Mar 19;41:102696. doi: 10.1016/j.pmedr.2024.102696 (PMC10997895; doi:10.1016/j.pmedr.2024.102696)
Supplement: Supplementary data 1 [file mmc1.docx]

**Supplementary Online Content**

***eMethods.*** Measurement of cognition & Personal SES estimations.

***eTable 1.*** CAIDE Dementia Risk Scores used in the study and number of points

***eTable 2.*** Cross-sectional correlation matrix of neighbourhood characteristics, socio-economic status, and education in the Healthy Brain Project sample, 2016-2020

***eReferences***

**eMethods**

*Measurement of cognition.*

The CBB consists of four tests. Detection (DET) is a simple reaction time task measuring psychomotor function, and Identification (IDN) is a choice reaction time task measuring visual attention. The primary outcome for DET and IDN was reaction time in milliseconds, with lower scores indicating faster completion time. The Attention Composite was computed by standardizing and averaging the DET and IDN tests. As DET and IDN are speeded measures, they were reverse-scored such that negative values reflected poorer performance. One Card Learning (OCL) is a continuous visual recognition task set within a pattern separation model, and One-Back (OBK) is a task of working memory. The primary outcome for OCL and OBK was the proportion of correct responses, normalized using an arcsine square-root transformation, with higher scores indicating better performance. The Memory Composite was computed by standardizing and averaging the OCL and OBK test scores. Tests were standardized using the baseline mean and standard deviation of the entire sample.

*Personal SES estimations.*

Two authors independently coded participants’ occupations into the Australian and New Zealand Standard Classification of Occupations (ANZSCO; ABS 2006), with any discrepancies resolved by consensus. Each occupation has a corresponding weighted AUSEI06 score.^1^ Where possible, responses were coded into the level of the ANZSCO minor groups, otherwise, a superordinate category was used (i.e., sub-major or major groups). Each occupational group has a corresponding AUSEI06 value which was used as the measure of personal SES. For participants who had retired, we coded the AUSEI06 score using their last occupation prior to retirement.

***eTable 1.*** ***CAIDE Dementia Risk Scores used in the study and number of points***

| Criteria | Original CAIDE Score (Points awarded) | Modified CAIDE Score (Points awarded) |
| --- | --- | --- |
| Age, years  <47  47-53  >53 | 0  3  4 | - |
| Education, years  ≥10  7-9  0-6 | 0  2  3 | - |
| Sex  Women  Men | 0  1 | - |
| Hypertension  SBP ≤140 mmHg  SBP >140 mmHg | 0  2 | 0  2 |
| BMI, kg/m^2^  ≤30  >30 | 0  2 | 0 2 |
| Hypercholesterinemia  ≤6.5 mmol/L  >6.5 mmol/L | 0  2 | 0 2 |
| Physical activity  Active  Inactive | 0  1 | 0 1 |
|  | Max 15 points | Max 7 points |

CAIDE: Cardiovascular Risk Factors, Aging and Dementia; SBP: systolic blood pressure; BMI: Body Mass Index. Components of Age, Education, Sex were scored using demographic data collected. Hypercholesterinemia was scored according to participant self-reports of high cholesterol diagnosis rather than quantitative blood work (without diagnosis substituted for ≤6.5 mmol/L and scored 0; with diagnosis substituted for >6.5 mmol/L and scored 2). Hypertension and BMI were also scored using self-report questionaries. Physical activity level was captured using the International Physical Activity Questionnaire (IPAQ4), with “High” or “Moderate” IPAQ activity levels corresponding to “Active” and scored 0; a “Low” IPAQ activity level corresponded to “Inactive” and was scored 1. The scores from each component were summed to acquire the CAIDE Dementia Risk total score. The modified CAIDE did not include age, sex, or education in the calculation of the scores.

***eTable 2.*** ***Cross-sectional correlation matrix of neighbourhood characteristics, socio-economic status, and education in the Healthy Brain Project sample, 2016-2020***

|  | Percentage Greenspace ^L2^ | | Distance to Greenspace ^L2^ | | Intersections per km^2^ | | Crime rate ^O.L2^ | | Population density | | IRSAD | | AUSEI06 | | Urban | | | | | | | |
| --- | --- | --- | --- | --- | --- | --- | --- | --- | --- | --- | --- | --- | --- | --- | --- | --- | --- | --- | --- | --- | --- | --- |
|  | *r* | *p* | *r* | *p* | *r* | *p* | *r* | *p* | *r* | *p* | ***r*** | ***p*** | ***r*** | ***p*** | | ***r*** | | | | ***p*** | | |
| Distance to Greenspace ^L2^ | **-0.476** | **<.001** |  |  |  |  |  |  |  |  |  |  |  |  | |  | | | |  | | |
| Intersections per km^2^ | **0.136** | **<.001** | **-0.610** | **<.001** |  |  |  |  |  |  |  |  |  |  | | |  | | | |  | |
| Crime rate ^O.L2^ | 0.152 | <.001 | **-0.497** | **<.001** | **0.407** | **<.001** |  |  |  |  |  |  |  |  | | |  | | | |  | |
| Population Density | -0.063 | .002 | **-0.388** | **<.001** | **0.909** | **<.001** | **0.309** | **<.001** |  |  |  |  |  |  | | |  | | | |  | |
| IRSAD | **0.080** | **<.001** | **-0.310** | **<.001** | **0.407** | **<.001** | **-0.141** | **<.001** | **0.370** | **<.001** |  |  |  |  | | |  | | | |  | |
| AUSEI06 | 0.020 | .181 | **-0.082** | **.001** | **0.103** | **<.001** | 0.018 | 0.280 | **0.108** | **.001** | **0.123** | **<.001** |  |  | | |  | | | |  | |
| Urban | 0.123 | <.001 | **-0.577** | **<.001** | **0.584** | **<.001** | **0.262** | **<.001** | **0.481** | **<.001** | **0.442** | **<.001** | **0.063** | **.002** | | | |  |  | | |  |
| Education | 0.024 | .136 | **-.104** | **<.001** | **0.208** | **<.001** | -.016 | .290 | **.165** | **<.001** | **0.161** | **<.001** | **0.449** | **<.001** | | | | **.090** | | | **<.001** | |

IRSAD = Index of Relative Socioeconomic Advantage and Disadvantage; AUSEI06 = Australian Socioeconomic Index 2006. Note, crime rate and intersections are based on data from the state of Victoria only.

**eReferences**

**1.** McMillan J, Beavis A, Jones FL. The AUSEI06: A new socioeconomic index for Australia. *Journal of Sociology.* 2009;45:123-149.
